# Variation in Anatomical Position of Vermiform Appendix among Iranian Population: An Old Issue Which Has Not Lost Its Importance

**DOI:** 10.1155/2014/313575

**Published:** 2014-09-10

**Authors:** Ahmad Ghorbani, Mehdi Forouzesh, Amir Mohammad Kazemifar

**Affiliations:** ^1^Department of Forensic Medicine, Ahvaz Jundishapur University of Medical Sciences, Ahvaz, Iran; ^2^Local Office of Iranian Legal Medicine Organization, Zenjan, Iran; ^3^Department of Internal Medicine, Qazvin University of Medical Sciences, Qazvin 34137 86165, Iran

## Abstract

Vermiform appendix has diverse anatomical positions, lengths, and conditions of mesoappendix. Knowing the exact anatomical position of vermiform appendix is important in view of surgeons for on-time diagnosis and management of acute appendicitis. The aim of present study is determination of these characteristics of vermiform appendix among Iranian population. The present study was conducted on 200 bodies, selected from the dead bodies that had been referred to local bureau of legal medicine, Zenjan province, Iran, for medicolegal autopsy since 21 Mar 2010 to 21 Mar 2011. According to the results, the anatomical positions of the appendix were pelvic, subcecal, retroileal, retrocecal, ectopic, and preileal in 55.8%, 19%, 12.5%, 7%, 4.2%, and 1.5% of the bodies, respectively. The mean length of vermiform appendix was 91.2 mm and 80.3 mm in men and women, respectively. Mesoappendix was complete in 79.5% of the bodies. No association was found between sex and anatomical position of vermiform appendix. Anterior anatomical position was the most common position for vermiform appendix. It is inconsistent with most related reports from western countries. It might be possible that some factors, such as race, geographical changes, and dietary habits, play roles in determining the position of vermiform appendix.

## 1. Introduction

Vermiform appendix is a part of the digestive tract which lies in right lower quadrant of abdomen. It has a worm-like structure and arises during embryological life from the posteromedial wall of the cecum, about 2 cm below the ileocecal valve [1]. The first comprehensive study made of the position of the appendix was completed by Gladstone and Wakeley in 1924, who studied 3000 anatomic dissections. Previous to this, other authors had stated their belief, from observations at necropsy or operation, that the majority of appendixes are situated anteriorly and that they are free and hang over the brim of the pelvis [1]. Though a remarkably constant structure in man, the appendix is nevertheless occasionally subject to the extremes of variation, that is, total suppression and duplicity. Its length varies from 2 to 20 cm, in average 9 cm [2]. The base of appendix is connected to the cecum, but its head can be placed in different situations. The diversity of situations is categorized into six locations: retrocecal, pelvic, subcecal, preileal, retroileal, and ectopic [3–6].

Acute appendicitis is the most common cause of acute abdomen among young patients. However it may also be seen in any age group [4]. Some studies have shown that age, race, sex, geographic region, and diet can affect the position of appendix [3]. Mortality rate of nonperforated appendicitis is 0.1% which is slightly higher than the mortality rate of a general anesthesia. But, the mortality rate in perforated appendicitis is about 3%, while it may be as high as 15% in the elderly population [4]. Dejanlić et al. have evaluated 65 patients who underwent open appendectomy in Serbia. They have reported pelvic position as the most common position for vermiform appendix (57.71%) and paracecal as the least one (3.07%) [7].

Acute appendicitis is mainly diagnosed by medical examination and clinical evaluation. There is no definitive diagnostic laboratory test or imaging [8]. Knowing common position(s) of the appendix helps on-time diagnosis of acute appendicitis. Variable positions of the appendix may mislead physicians to make a wrong decision or diagnosis of other diseases. Delayed diagnosis of acute appendicitis may lead to its perforation and subsequent abscess or peritonitis. So, accurate information about the anatomical location of appendix can improve prognosis of the disease.

The present study was performed to determine the anatomical locations of the appendix, its length and span, and status of mesoappendix of appendix (complete or incomplete), as well as their relationship with age and sex on the dead bodies that had been referred for medicolegal autopsy.

## 2. Methods and Materials

The present study was conducted on 200 bodies (153 males and 47 females). They were randomly selected from the dead bodies that had been referred to local bureau of legal medicine of Zenjan province, Iran, for medicolegal autopsy since 21 March 2010 to 21 March 2011. We selected 200 cadavers which had been referred to forensic autopsy center: 153 of them were male and 47 were female. The study population was randomly chosen from cadavers that needed autopsy to determine their cause of death. They were from different ages and sexes. The inclusion criteria were Iranian citizen and necessity to perform autopsy. Unknown cadavers, non-Iranian cadavers, cadavers with severe burns, disintegrated cadavers, decomposed cadavers, cadavers with congenital anomalies, old or new abdominal surgery, peritonitis, intestinal distension, and any reason which might change the anatomical position of the appendix were excluded from the study. Anatomical location of the appendix was determined during autopsy. Appendix length was measured in millimeters with a ruler and completeness of mesoappendix was determined by one of the authors. Sex of the bodies was determined based on the observed phenotype, and their age was recorded based on their identity document. The collected data was analyzed using SPSS software version 16.0. We used descriptive parameters and chi-square test for analysis and reporting of the results.

This study was approved by the ethical committee of local bureau of legal medicine of Zenjan province. No additional incisions were made in the skin of the bodies for conduction of the study.

## 3. Results

This study was done on 200 bodies: 153 of them (76.5%) were male and 47 (23.5%) were female. The mean age of the study population was 39.3 years.

According to the results, anatomical locations of the appendix were as follows: pelvic in 111 individuals (55.8%), subcecal in 38 individuals (19%), retroileal in 25 individuals (12.5%), retrocecal in 14 individuals (7%), ectopic in 9 individuals (4.2%), and preileal in 3 individuals (1.5%). The most common anatomical location was pelvic location for both sexes. Subcecal, retroileal, retrocecal, ectopic, and preileal locations were in the following places in both sexes, respectively. No anatomical position of the preileal was observed in female population.

The minimum length of appendix was 15 millimeters and its maximum length was 175 millimeters. The average length of appendix was 91.2 mm for men and 80.3 mm for women. When appendix was classified according to its length, the most individuals lie in length range from 80 to 119 mm (Table 1).

In this study, a significant association was found between the appendix length and different age groups (*P*  value < 0.001). The details are shown in Table 2. The highest length of appendix was seen in people 11–19 years old. Appendix length was significantly greater in men (*P*  value = 0.01).

Mesoappendix was complete in 159 (79.5%) of the studied sample. Incomplete mesoappendix was mostly seen in the age group below 10 years. There was not any significant relationship between gender and status of mesoappendix (*P*  value = 0.30).

## 4. Discussion

In our study, the most common position of appendix was pelvic position and the lowest was preileal position. Our findings were similar to studies of Katzurski et al. [9], Ojeifo et al. [10], Rahman et al. [11], and Paul et al. [12]. However, there are some different findings in other studies. L. Ajmani and K. Ajmani in India [13], Ojeifo et al. in Bosnia [10], and Clegg-Lamptey et al. in Ghana [14] have reported that the most common position of appendix is retrocecal and pelvic. It seems that many factors, including race, are involved in determining the position of the appendix. Our findings were similar to studies of Denjalić et al. [7] and Golalipour et al., conducted in Iran [15], who have evaluated the patients in surgery ward, study of Yabunaka et al. [16] who have evaluated the size of appendix by sonography, and study of Ahmed et al. [17] who have estimated the appendix size during therapeutic laparotomy. The most common position of appendix has been pelvic position in all of these studies. Variable positions of vermiform appendix may have an effect on the diagnosis of appendicitis, which is one of the most common causes of acute abdomen [18].

If the location of appendix was viewed in relation to the cecum, it can be divided into anterior (pelvic and pre- and retroileal) or posterior (retrocecal and paracecal) locations [8]. The anterior location of appendix was seen in more than 75% of our studied population. So, early diagnosis of appendicitis and shorter duration of surgery and hospitalization are expected among Iranian patients. This can reduce the complications of appendicitis surgery [8].

Pelvic position is the most common location of the appendix in both males and females. Its frequency was 32 (67%) in female population and 80 (52.2%) in male population. It indicates that pelvic location is most frequently seen in females. On the other hand, it has been hypothesized that the vermiform appendix is a mobile organ whose position may change with various situations [6].

In our study, the length of the appendix was longer in men than in women. It is similar to studies of Katzurski et al. [9], Gholalipour et al. [15], and L. Ajmani and K. Ajmani [13], but contrary to studies of Bakheit and Warille [19] and Rahman et al. [11] that have reported that length of the appendix in women is longer than in men. Searle et al. believes that after an initial growth period during early infancy up to about 3 years, the appendix achieves its adult proportions and does not continue to grow throughout childhood [20]. However, we found that individuals with older age have longer length of the appendix.

We showed that frequency of incomplete mesoappendix is highest in the age group below 10 years. Incomplete mesoappendix may reduce blood supply to the tip of the appendix and make it prone to gangrene and perforation. It may imply poor outcome of acute appendicitis in children. Incomplete mesoappendix could be one of the reasons of the severity of appendicitis in childhood.

In conclusion, high incidence of anterior position and complete mesoappendix in our population entail that diagnosis of acute appendicitis could be sooner and easier in our population and its complications, such as perforation and gangrene, might be less than other populations. Also, the duration of open or laparoscopic surgery and patients' hospitalization are expected to be reduced.

## Figures and Tables

**Table 1:** Frequency (percent) distribution of appendix length among studied people.

Length of appendix	<40 mm	40–79 mm	80–119 mm	>119 mm	Total

Frequency (%)	3 (1.5)	63 (31.5)	101 (50.5)	33 (16.5)	200 (100)

**Table 2:** Association between length of appendix and age among studied people.

Length of appendix (mm)						
	<40	40–79	80–119	>119	Total	*P* value
Age (y/o)						
<10	1 (0.5%)	9 (4.8%)	2 (1%)	1 (0.2%)	13 (6.5%)	**<0.001**
11–19	0	4 (2%)	2 (0.8%)	3 (1.8%)	9 (4.5%)	**<0.001**
20–39	1 (0.5%)	27 (13.8%)	50 (24.8%)	12 (6.2%)	91 (45.3%)	**<0.001**
40–54	0	10 (5%)	20 (10.2%)	9 (4.2%)	39 (19.5%)	**<0.001**
>55	1 (0.5%)	12 (6%)	27 (13.8%)	8 (4%)	49 (24.2%)	**<0.001**

Total	**3 (1.5 %)**	**63 (31.5%)**	**101 (50.5%)**	**33 (16.5%)**	**200 (100%)**	**<0.001**
